# Professor Lizars

**Published:** 1860-09

**Authors:** 


The Late Professor Lizars.—In our last issue we announced the death of this distinguished surgeon, and promised our readers a short sketch of his life and medical services.

Dr. John Lizars was born in Edinburgh, and obtained his degree in 1808. He was the pupil of John Bell, and soon after his graduation he entered the royal navy, and held the post of surgeon for several years. Seven years later, he returned to Edinburgh, and was admitted into partnership with John Bell and Robert Allan, and began a course of lectures on anatomy and physiology; but in a few years he lectured on anatomy alone, and several years subsequently he also took up surgery. In 1831, Dr. Lizars received the appointment of Professor of Surgery to the Royal College of Surgeons of Edinburgh, and was also appointed Surgeon to the Royal Infirmary.

To Dr. Lizars is erroneously ascribed the operation of extirpation of the superior maxilla. The honor, however, of introducing this procedure is due to the late Dr. Jameson, of Baltimore, who, in 1820, was the first to remove the whole of the bone. Dr. Lizars was a popular lecturer, and a cool and dextrous surgeon. In fact, as an operator, he could not be surpassed. His contributions to anatomy and surgery are numerous; and he is better known by his "System of Practical Surgery," and the "Anatomical Plates." Besides these works, he wrote on the "Extraction of Diseased Ovaria," on "Stricture," on "Strabismus," and on "Club-foot."

